# Unleashing endogenous TNF-alpha as a cancer immunotherapeutic

**DOI:** 10.1186/s12967-018-1611-7

**Published:** 2018-08-31

**Authors:** Steven F. Josephs, Thomas E. Ichim, Stephen M. Prince, Santosh Kesari, Francesco M. Marincola, Anton Rolando Escobedo, Amir Jafri

**Affiliations:** 1 Immunicom, Inc., San Diego, CA USA; 2 Immune Advisors LLC, La Jolla, San Diego, CA USA; 3 John Wayne Cancer Institute and Pacific Neuroscience Institute, Santa Monica, CA USA; 4 CHIPSA Hospital, Baja California, Mexico

**Keywords:** Immunotherapy, TNF-alpha, Extracorporeal, Soluble TNF-alpha receptors, Cancer, Immuno-oncology

## Abstract

Tumor necrosis factor (TNF)-alpha was originally identified in the 1970s as the serum mediator of innate immunity capable of inducing hemorrhagic necrosis in tumors. Today, a wide spectrum of biological activities have been attributed to this molecule, and clinical translation has mainly occurred not in using it to treat cancer, but rather to inhibit its effects to treat autoimmunity. Clinical trials utilizing systemic TNF-alpha administration have resulted in an unacceptable level of toxicities, which blocked its development. In contrast, localized administration of TNF-alpha in the form of isolated limb perfusion have yielded excellent results in soft tissue sarcomas. Here we describe a novel approach to leveraging the potent antineoplastic activities of TNF-alpha by enhancing activity of locally produced TNF-alpha through extracorporeal removal of soluble TNF-alpha receptors. Specifically, it is known that cancerous tissues are infiltrated with monocytes, T cells, and other cells capable of producing TNF-alpha. It is also known that tumors, as well as cells in the tumor microenvironment produce soluble TNF-alpha receptors. The authors believe that by selectively removing soluble TNF-alpha receptors local enhancement of endogenous TNF-alpha activity may provide for enhanced tumor cell death without associated systemic toxicities.

## Background

The history of TNF-alpha is very closely related to the history of tumor immunotherapy. In the early 1900s, the New York physician William Coley observed that various cancer patients would enter remission after experiencing bacterial infections. In a brave set of experiments, Dr. Coley began emulating bacterial infections by purposely administering various combinations of pathogens to patients. One of these mixtures, containing *Streptococcus pyogenes* and *Serratia marcescens*, was demonstrated to possess therapeutic activity and became widely used in the USA prior to the advent of chemotherapy and radiotherapy. Today, such “Coley’s toxins” are limited due to lack of controlled clinical trials and FDA approvals. In the 1960s, attempts to identify the molecular mechanisms by which Serratia marcescens induced tumor regression led to the discovery of a “factor” in the sera of treated mice [1]. This factor was identified in 1975 as “Tumor Necrosis Factor” (TNF-alpha) [2]. It was found that this endotoxin induced factor, was also inducible with known immune stimulants such as bacillus Calmette–Guerin (BCG), zymosan, and Corynebacteria. The isolated factor had the capability to directly kill tumor cells in vitro, but there were no deleterious effects on proliferating non-malignant murine embryonic cells.

Molecular analysis led to cloning of the cDNA and revealed the molecule was comprised of 233 amino acids with a leader sequence of the first 76 amino acids [3, 4]. Interestingly, it was found that the same sequence belonged to another factor associated with cancer: Cachectin [5]. Cachectin was originally demonstrated to mediate weight loss and alter normal metabolic priorities through its effects on both the central nervous system (CNS) and peripheral tissues. Early studies showed that administration of cachectin in animals induces cachexia with a pattern of tissue wasting that includes whole-body protein depletion, unlike the protein-conserving pattern induced by simple caloric restriction [6, 7]. Given the inflammatory nature of TNF-alpha, studies where performed to assess its role in endotoxin-induced shock models.

It was found that administration of TNF-alpha in quantities approximating endogenous levels that were observed in response to endotoxin resulted in hypotension, metabolic acidosis, hemoconcentration, and death due to respiratory arrest within minutes to hours. This sequence resembled sepsis associated symptomology. Hyperglycemia and hyperkalemia were also observed after infusion. At necropsy, diffuse pulmonary inflammation and hemorrhage were apparent on gross and histopathologic examination, along with ischemic and hemorrhagic lesions of the gastrointestinal tract, and acute renal tubular necrosis [8–11]. Thus TNF-alpha, appeared to not only be a potent mediator of tumor regression, but also an effector of cachexia, and a contributor to one of the main mechanisms leading to septic shock.

## TNF-alpha forms and family

TNF-alpha is found in a soluble and membrane bound form. The soluble plasma form of TNF-alpha is cleaved from the membrane forms by a metalloproteinase termed TNF-alpha-converting enzyme (TACE) which belongs to the ADAMs family of disintegrins [12, 13]. Soluble TNF-alpha is 17-kDa protein consisting of 157 amino acids that forms a homotrimer for receptor activation. TNF-alpha is mainly produced by activated macrophages, T lymphocytes, and natural killer (NK) cells [14]. A related but distinct cytokine, TNF-beta, previously known as Lymphotoxin was characterized to share some of the activity of TNF-alpha [15–17]. At present count, there are 19 members of the TNF family and 29 receptors that have been characterized [18–20].

## TNF-alpha receptors 1 and 2

The activity of TNF-alpha is mediated through two cell surface receptors, TNF-R1 (p55) and TNF-R2 (p75) that differ in their signaling activity. TNF-R1 is usually pro-apoptotic whereas TNF-R2 is usually anti-apoptotic [21]. TNF-R1 and TNF-R2 have similar extracellular TNF-binding structures characterized by four repeated cysteine-rich domains but have different intracellular domains [22]. The main structural difference between TNF-R1 and TNF-R2 that accounts for their divergent biological activity resides in that TNF-R2 lacks an intracellular death domain. Thus, in many systems, TNF-alpha promotes apoptosis through activating TNF-R1 but causes pro-survival signaling through TNF-R2 [23–28]. After binding TNF-alpha, TNF-R1 recruits the adaptor protein TNF-R1-associated death domain protein (TRADD) and its downstream caspases (i.e. Caspase 8) causing apoptosis [18, 29, 30]. Conversely, when TNF-alpha activates TNF-R2, recruitment of the TNF receptor-associated factors (TRAF2) occurs, resulting in stimulation of NF-kappa B, which possesses anti-apoptotic properties [19, 31]. TNF-R1 is the high affinity receptor which is internalized upon ligation whereas TNF-R2 is shed [32]. TNF-R2 is known to possess a higher affinity towards membrane bound TNF-alpha as compared to soluble TNF-alpha [33].

While TNF-R1 is expressed on various tumor cells [34], and tumor endothelial cells [35], TNF-R2 is expressed on various immune cells including T regulatory cells [36, 37], myeloid suppressor cells [38], and some cancer cells [39, 40]. That TNF-R1 receptor is mainly responsible for the toxicity is demonstrated by its reduction by treating with antisense TNF-R1 [41]. Tumor resistance to the cytotoxic effects of TNF-alpha is mediated by TNF-R2. For example, in the Lewis Lung Model, knock down of TNF-R2 in the cancer cells promotes robust anti-tumor effects upon administration of low dose murine TNF-alpha whereas in wild type mice it enhanced tumor growth while the TNF-R1 knockdown was not affected [42]. Moreover, TNF-R2 activation has been implicated in T-reg expansion and immune-suppression [28, 43].

## Role of TNF receptors in cancer

The effects of systemically administered TNF-alpha are blocked by soluble receptors, sTNF-R1 and sTNF-R2, that are released into the plasma [44]. These are cleaved from the membrane forms by TACE (ADAM17) upon the introduction or release of soluble TNF-alpha [45–47]. The receptor ligand affinity is largely dependent on the adaptor protein recruitment [48]. However TNF-alpha mutants have been developed that specifically bind either TNF-R1 or TNF-R2 [49] and novel mutants have been obtained with lower toxicity and increased anti-tumor activity compared to wild type TNF-alpha [50].

The concentration of the soluble receptors increases following exposure to TNF that is produced after infections or upon administration of recombinant TNF as a function of the natural TNF buffering system to control runaway cytokine response [47]. These receptors have been found to be elevated in tumors and in the plasma of cancer patients [51] as a mechanism of tumor survival by counteracting the anti-cancer potential of TNF-alpha [52, 53]. Various complex interplays between receptors have been described based on vitro studies, which in some cases are contradictory. Accordingly, we will discuss below the cellular effects of TNF-alpha in various immunological and cancer systems.

## Cellular effects of TNF-alpha

**Fig. 1:**
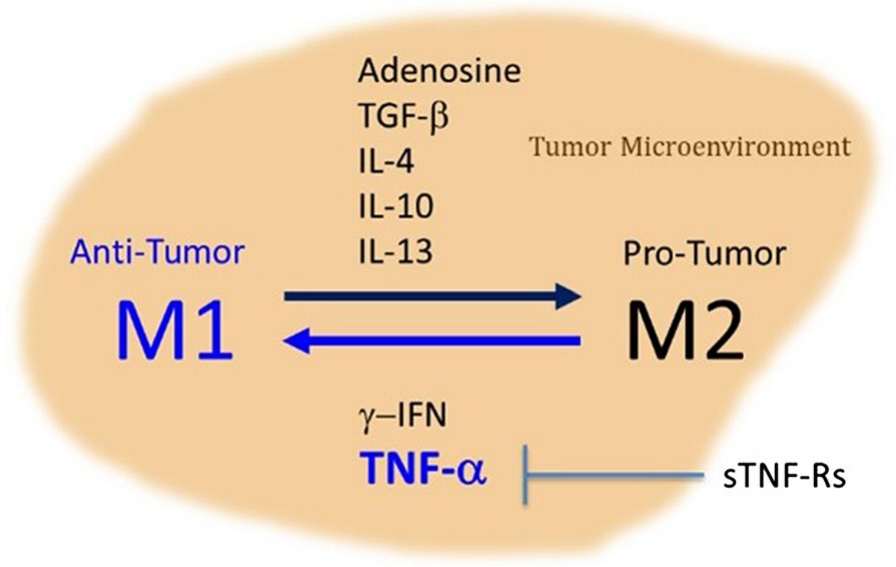
Turning Cold Tumors Hot. TNF induces inflammation and anti-tumor M1. Soluble TNF receptors block the effects of TNF. Removal of sTNF-Rs unleashes TNF activity

Approximately 28% of cancers are susceptible to direct cell killing mediated by soluble TNF. The anti-tumor activity of TNF-alpha is now well established and can be mediated through a variety of mechanisms including: (1) Cellular apoptosis by binding to tumor cell surface receptors; (2) T-effector cell activation (macrophage and NK cells) by blocking T-Reg cells that are immune suppressors [54, 55]; (3) Inducing tumor microvasculature collapse through endothelial cell modulation and disruption of neoangiogenesis including disruption of tumor vasculature [56, 57]; (4) Promoting TAM (tumor associated macrophages) to M1 anti-tumor stage (see Fig. 1); (5) Attraction and stimulation of neutrophils and monocytes to sites of activation for anti-tumor immune responses [58, 59]; and (6) Downregulation of IL-13 expression by eosinophilic-like cells and inhibition of tumor induced monocyte differentiation to immunosuppressive phenotypes [60].

As the most pleiotropic of cytokines, TNF-alpha contributes to both inflammation during infections and anti-inflammatory/tissue repair processes after clearance of infections [61]. Its effects at low levels of expression contribute to tumorigenesis [62]. Upon exposure to inflammatory stimuli, TNF-alpha as well as IL-1 and other chemokines are produced mainly by activated macrophages and other cells of the myeloid lineage which attracts and activates neutrophils and monocytes to the tumor site. In tumors, the membrane form of TNF-alpha on tumor cells interacts mainly with TNF-R2 to activate clearance of pro-tumor suppressor cells via the production of reactive oxygen intermediates with signaling through ceramide [63]. Consequently, intratumoral preservation of the membrane form (with lower intratumoral TACE activity) is associated with better prognosis [64]. Reverse signaling can also occur where the receptors can activate intracellular processes after binding to the membrane form of TNF-alpha [65]. Neither TNF-alpha nor its receptors are lethal in murine knockout models. However, the organization of the lymphoid organs and thymus function are affected [66].

The anti-tumor effects of TNF-alpha have been demonstrated on primary tumors with significant pancancer effects through vascular destruction and tumor necrosis [56]. This effect is mainly attributable to TNF-R1 ligation. However, minimal residual disease (MRD) may be stimulated upon infections and lead to the development of resistance to cytotoxic drugs [67]. In a murine model, treatment with anti-TNF antibodies delays the onset of recurrence after initial complete regression of primary tumors. In attempt to control the potential pro-tumor effects of TNF-alpha, human clinical trials were conducted using anti-TNF antibodies or receptors and these have met with limited disease stabilization in approximately 20% of the patients [68–70]. However, induction of lymphoma or skin cancer was also observed [71, 72]. The ability to specifically block the anti-inflammatory/tissue repair processes of TNF-alpha in MRD would be of significance with respect to controlling the recurrence of tumors. An elegant approach would be to identify methods with cytokine or drug combinations that induce long-term immune responses such as the combination of TNF and gamma Interferon [73]. Obviously, predicting outcomes will remain speculative until clinical trials are conducted.

## Potential for general anti-tumor therapy via vascular disruption by TNF

Tumors can be categorized into three major types regarding their response to chemotherapy immune modulators: “hot”, “cold” and “intermediate”. Hot tumors contain a plethora of cell infiltrates whereas cold tumors have relatively few. Intermediate tumors are types that fall in between. Clinically, hot tumors tend to respond well to therapy, whereas cold tumors are resistant. Intermediate tumors may respond at first but then become resistant to therapy.

With few exceptions, tumors are dependent on neovascularization and in theory share a common susceptibility to TNF induced vascular disruption. In sufficient amounts, the global effect of TNF is predictably rapid, dependent on pertussis toxin G-protein inhibition and stimulation of the release of protein S from the tumor endothelium to promote vascular modulation, and induces fibrin accumulation with clotting and enhancement of permeability leading to necrosis [74]. This effect is dependent on C5a complement factor [75]. Of interest is that specific delivery of TNF-alpha to tumor sites promotes anti-tumor effects [76, 77]. Overexpression of TNF in cancer cells results in longterm tumor growth suppression, independent of IL-12 or IL-18 and works via a STAT1 and IFN regulatory factor 1-dependent IFN-gamma pathway [78]. Such higher-than-physiologic concentrations of TNF work through similar mechanisms in the normal vasculature leading to systemic toxicity. Factors in the tumor microenvironment contribute to the greater sensitivity of the tumor vasculature to TNF.

## Clinical trials of TNF-alpha for cancer therapy

Initial clinical trials of TNF-alpha utilized systemic administration. Phase 1 studies all reported sepsis-associated symptoms as dose-limiting toxicities [79]. For example, Kimura et al. administered intravenous infusions starting at 1 × 10 [5] units/m^2^ and escalated to 16 × 10 [5] units/m^2^. Fever, rigors, nausea and vomiting, and anorexia toxicities where found to be non-dose-dependent; whereas hypotension, leukocytosis, thrombocytopenia and transient elevation of transaminases (SGOT and SGPT) where dose-dependent. Disseminated intravascular coagulopathy (DIC), a classical symptom of sepsis, was observed at the highest dose. The authors concluded that the maximum tolerated dose was 12 × 10 [5] units/m^2^ [80]. Other studies found similar toxicities associated with systemic TNF-alpha administration, with little or no favorable achievement in tumor response [81–84]. Part of the cause of TNF-alphaassociated systemic toxicity is its ability to induce alterations to endothelial cells, resulting, in part, in augmentation of coagulopathy [85].

Given the inability to translate the profound anti-tumor effects observed in animal studies to human studies, some researchers have explored localized administration of TNF-alpha in hopes of avoiding adverse effects associated with systemic use. One of the first reports describing localized administration of TNF-alpha was Kahn et al. who treated 27 patients suffering from Kaposi’s sarcoma. Intratumoral administration reduced the cross-sectional area in 15 of 16 injected cancer lesions and caused the complete disappearance of three lesions [86]. The noted high degree of vascularization in Kaposi’s sarcoma may be one of the explanations for the high degree of success, given that TNF-alpha is known to induce vascular hemorrhage in malignant tissues [87–89]. Multiple mechanisms are believed to be associated with tumor vascular damage by TNF-alpha, including induction of release of von Willebrand Factor, which is a known anti-coagulant, as well as endothelial cell activation leading to thrombosis [90].

One clinically successful utilization of localized TNF-alpha therapy is its use (usually in combination with the alkylating agent melphalan) in isolated limb perfusion (ILP) protocols. Early studies demonstrated synergy between TNF-alpha and melphalan in animal models of ILP [91, 92]. These procedures have been translated to patients with melanoma and soft tissue sarcomas, where the complete response rate has been 80%. It is believed that there are two mechanisms by which TNF-alpha functions as a contributor to such high response rates. The first involves augmentation of endothelium permeability, which facilitates entrance of chemotherapy, and the second mechanism involves direct killing of tumor endothelium, which results in vascular leakage. Hemorrhagic necrosis of tumors is observed in a rapid manner subsequent to TNF-alpha and melphalan administration, with disruption of cell–cell adhesive junctions occurring within minutes, followed by tumor vascular collapse 24 h later [58, 93]. From a clinical perspective TNF-alpha ILP therapy was approved in Europe for high grade soft tissue sarcoma in 1998 [94].

Another clinical success of localized TNF-alpha administration is in liver metastasis where isolated hepatic perfusion (IHP) may be performed. IHP was first clinically applied almost 50 years ago [95] and offered the ability to locally administer high concentrations of chemotherapeutic agents without systemic toxicities. In extending IHP to TNF-alpha administration, one phase 2 trial at the Surgery Branch of the NCI, the overall response rate in 50 patients was reported at 74% and was observed across virtually all types of histologies treated. The response rates were maintained even in patients who had numerous metastases, large metastases, or who had a significant percentage of liver replaced by tumor. Overall the duration of response was 9 months, although in some patients it was more than 3 years [95]. To assess the contribution of TNF-alpha versus melphalan alone, 22 patients with ocular melanoma metastatic to liver were treated: 11 with melphalan alone, and 11 with TNF-alpha and melphalan. Patients possessed advanced tumor burden with a mean percentage of hepatic replacement of 25%. The overall response rate in 21 patients was 62% including 2 radiographic complete responses (9.5%) and 11 partial responses (52%). The overall median duration of response was 9 months (range 5–50) and was significantly longer in those treated with TNF than without (14 versus 6 months, respectively). This study points to the importance of TNF-alpha in isolated perfusion protocols [96]. Interestingly, in animal models of IHP, correlations are seen between degree of tumor vascularization and tumor reduction, further suggesting that TNF-alpha mechanistically targets the tumor endothelium [97].

## Extracorporeal removal of soluble TNF-alpha receptors as a therapeutic

In the light of their pro-tumor activity, the removal of soluble TNF-Rs seemed to be a logical step toward the development of an effective anti-cancer therapy [52, 53]. To test the anti-cancer effects of removing inhibitory sTNF-Rs, Immunicom, Inc. conducted a preclinical canine cancer study using a novel single-chain TNF-alphabased affinity column (a.k.a. “LW-02” device) used in combination with a Terumo Optia apheresis system. The blood from catheterized canine patients was pumped into the Optia system which separated the patients’ plasma from their cells by continuous centrifugation. During each treatment, an LW-02 affinity column device was placed into the plasma flow line to capture sTNF-Rs from the patient’s plasma which was then recombined with the previously separated cells and returned to the patient. Most of the dogs in the trial were stage III or IV patients which had failed standard therapeutic approaches. The results of the study were very encouraging.

Overall, 50–60% of the patients treated were observed to have either stable disease or partial responses by RECIST Criteria during treatment with one patient having experienced a complete response with clearance of metastases. In over 300 treatments, throughout the study, the LW-02 devices appeared to be safe with no adverse events that were attributable to their use. The quality of life of the patients was effectively maintained during the treatment regimens and a significant life extension was observed based on initial prognoses.

In humans, few treatment options are available for stage IV patients that are unresponsive to standard treatments. Thus, subtractive therapies may be an effective alternative to fill this gap.

There is also potential for its use: (1) In combination therapies with cytotoxic drugs that induce or are enhanced in combination TNF-alpha (Table 1); (2) With immunotherapeutics such as antibodies to PD1, PDL1 or CTLA4; and/or (3) In combination with cytokines, such as IFNg and TNF-alpha [73]. It is speculated that the removal of TNF receptors may increase the effectiveness of TNF-alpha administration while decreasing systemic toxicity.

**Table 1:** Table of cytotoxic drugs that induce TNF-alpha and are enhanced in their activity with TNF-alpha

Anti-neoplastic drug	Class	Enhanced with TNF-alpha
Paclitaxel (taxol)	Diterpene	Yes
Doxorubicin	Anthracycline	Yes
Cytoxan	Cyclophosphamide	Yes
Gemcitabine	Nucleoside analogue	Yes
Cytarabine	Nucleoside analogue	Yes
Melphalan	Nitrogen mustard	Yes

## Conclusion

TNF-alpha is a fundamental molecule in various aspects of immunology. Original efforts at therapeutic applications failed due to systemic toxicities. In the new era of cancer immunotherapy, a promising research direction is augmentation of endogenous TNF-alpha activity through removal of its soluble receptors.

## Abbreviations

CTLA-4: cytotoxic T lymphocyte antigen-4
IFN: interferon
IL-6: interleukin-6
ILP: isolated limb perfusion
PD-L1: programmed death ligand-1
